# A Threat to the Occident? Comparing Human Values of Muslim Immigrants, Christian, and Non-religious Natives in Western Europe

**DOI:** 10.3389/fsoc.2020.538926

**Published:** 2020-10-23

**Authors:** Christian S. Czymara, Marcus Eisentraut

**Affiliations:** ^1^Faculty of Social Sciences, Goethe University Frankfurt am Main, Frankfurt, Germany; ^2^GESIS - Leibniz Institute for the Social Sciences, Cologne, Germany

**Keywords:** immigration, Muslims, Christians, human values, Europe, religion, integration, natives

## Abstract

With a growing Muslim population, many European countries need to integrate Muslims into their societies. One aspect that can hinder successful integration are substantial differences in human values. This is because such values are consequential for attitudes as well as behavior. We compare basic human values between Muslim immigrants and non-Muslim natives in four European countries with distinct immigration histories and integration politics: Belgium, France, Germany, and Sweden. For most insightful comparisons, we contrast values of Muslim immigrants with those of Christian natives as well as those of non-religious natives. We employ data of more than 50,000 individuals based on the first eight waves of the European Social Survey. Our findings reveal significant differences in value priorities between Muslims, Christians and non-religious individuals in all four countries. Amongst other things, Muslim immigrants score particularly high in *conservation* values (*security* and *tradition/conformity*). At the same time, they also score higher in *self-transcendence* values (*benevolence* as well as *universalism*). While many of these findings are in line with theory and previous research, the higher score in *universalism* is unexpected. A potential explanation is the combination of religious traditionalism and discrimination experiences. In other words, religious traditions are associated with more conservative views, but being subject to marginalization can still result in an appreciation of equal opportunities. We find only limited support for differences in *hedonism*. Religiosity correlates with values of *tradition/conformity* for Muslim immigrants as well as for Christian natives. Thus, accounting for religiosity renders differences in these values between Muslims and other groups statistically insignificant. While most of these findings hold in all countries, differences are most pronounced in Sweden and lower in the other three countries, which is also true after accounting for differences in socio-economic status and religiosity between the three groups. This suggests that a combination of a country's history of diversity and national integration policies either encourages the convergence of values or leads to a solidification of value differences between groups. We discuss these political and social implications of our findings.

## Introduction

Europe is becoming increasingly diverse in terms of religion and ethnicity, including a strong increase of Muslims (Pew Research Center, 2017). Some natives view Muslim fundamentalism as a threat to liberal achievements of Western societies (Helbling, 2014). Some far-right politicians take up public concerns to justify restrictive migration policies (e.g., Kaminski, 2015; Waterfield, 2015). This dynamic does not only influence public opinion (Czymara, 2019) but also gives rise to hate crimes targeted against Muslims, especially after Islamist terror attacks (Borell, 2015). At the same time, some native Europeans consider Muslim fundamentalism as threatening secular norms (Helbling and Traunmüller, 2018). Existing public opinion data suggest that Muslims, on average, score higher on, for example, homonegative (van Den Akker et al., 2013; Jäckle and Wenzelburger, 2015) or Anti-Semitic (Kaplan and Small, 2006; Bevelander and Hjerm, 2015) views compared to other religious groups, and are more likely to hold patriarchal values (Alexander and Welzel, 2011) as well as dismissive attitudes toward gender equality (Diehl et al., 2009). Higher levels of traditionalism (Connor, 2010) or even fundamentalism (Koopmans, 2015) might be an explanation. It seems reasonable to assume that these intertwined conflicts between Muslims, Christians and non-religious groups in Europe lead to differences in worldviews.

We know little, however, about the distribution of *human values* of Muslim immigrants in Europe. Human values can either foster or impede the integration of immigrants because (dis-)agreement in worldviews in terms of values is typically regarded consequential regarding peaceful cohabitation within societies. Moreover, human values of natives are directly linked to the perception of immigration and the evaluation of different minorities (Davidov et al., 2019; Eisentraut, 2019). This lack of evidence is striking given that human values are consequential regarding, for example, attitudes toward homosexuality (Kuntz et al., 2015), toward immigration (Davidov and Meuleman, 2012; Eisentraut, 2019) or toward the welfare state (Kulin and Meuleman, 2015), as well as individual well-being (Burr et al., 2011) or behavioral aspects such as alcohol consumption (Schwartz et al., 2001). Previous studies have shown that human values in European societies differ, for example, across age groups (Robinson, 2013) or, to lesser degree, with sex (Schwartz and Rubel, 2005). However, Schwartz and Rubel (2005) report that most variation in human values is not explained by such demographics but by “culture,” understood as differences across countries. We exceed the rather vague culture definition of Schwartz and Rubel (2005) by comparing basic human values across religious and non-religious groups in several European countries. We examine human values of Muslim immigrants in Europe, a group that received only limited attention in the human values literature so far with, first, Christian natives and, second, non-religious natives. The comparison of these three groups will lead to insights into the interplay of immigration and religion in the process of value formation in Europe. This is because, in case Muslim immigrants and Christian natives share more values compared to non-religious natives, differences would likely stem from religiosity in general. If, on the other hand, Christian and non-religious natives hold more similar values, general religiosity could be ruled out as an explanation, pointing to specific effects of religion, minority status or carry-over effects of origin societies (Röder, 2015; Soehl, 2017).

We test these considerations based on more than 50,000 individuals contained in the European Social Survey. For further insights, we distinguish differences between groups within four European countries with distinct immigration histories and integration politics (Belgium, France, Germany, and Sweden). Moreover, we examine how far potential value differences between groups can be attributed to differences in each group's level of religiosity or to structural differences in socio-economic characteristics. Given the strong correlation between human values and both attitudes and behavior, conflicting values between social groups impede integration into host societies, ultimately threatening social cohesion within and across European countries.

## Basic Human Values and Religion

**Figure 1:**
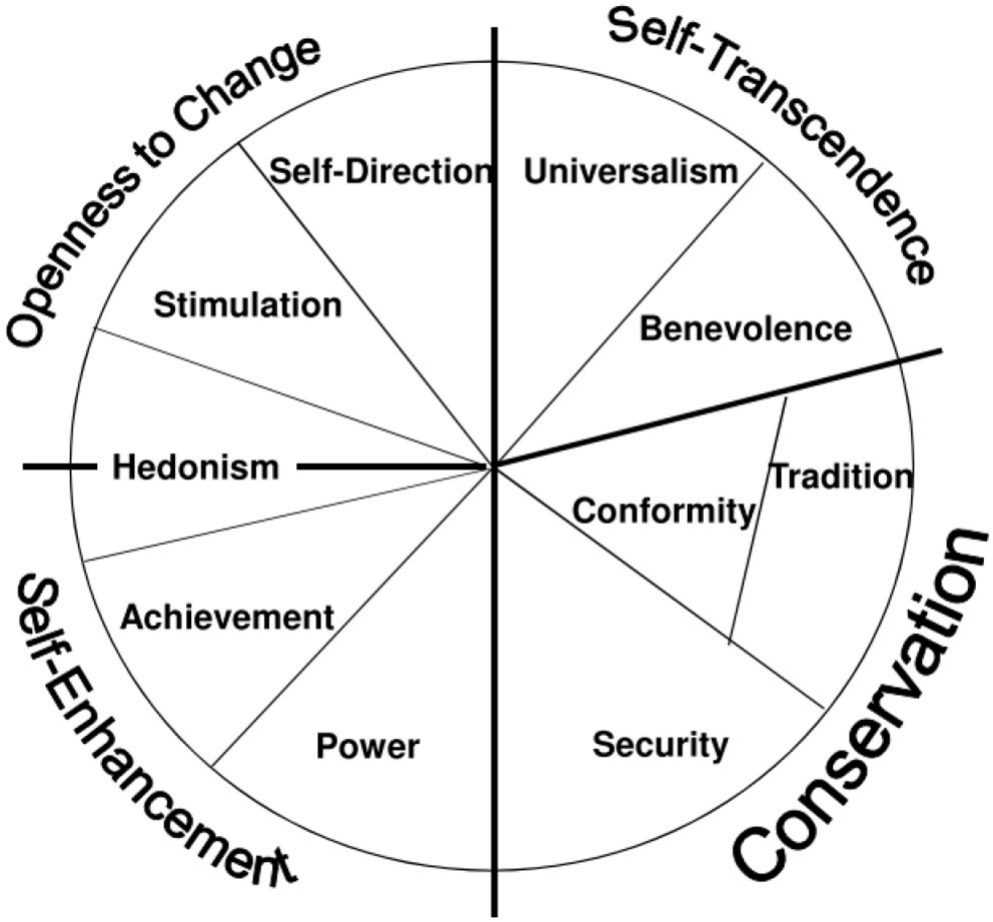
Basic human values according to Schwartz (2012).

Shalom H. Schwartz defines basic human values as “*desirable transsituational goals, varying in importance, that serve as guiding principles in the life of a person or other social entity”* (Schwartz, 1994, p. 21). Their importance is ordered in individual hierarchies that are usually viewed as (more or less) stable across time and situations (Rokeach, 1973). Therefore, individuals' values serve as guidelines to judge people, events, and actions. A plethora of studies has empirically validated the structure and definition (Schwartz, 1994) of basic human values (e.g., Schwartz and Boehnke, 2004; Davidov et al., 2008; Schwartz et al., 2012; Steinmetz et al., 2012). Figure 1 displays the quasi-circumplex structure of the basic human values. Adjacent values share common motivational cores and are, thus, more compatible with each other, whereas conflicting values and incompatible motivational goals are located on opposing sides of the circle. Each of the 10 values belongs to one of the four higher-order dimensions, which have two different lines of conflict. The first set of values we are interested in involve avoidance of change, self-restriction and order. These values are subsumed under the broader category *conservation*, in contrast to values expressing the need for new experiences (*openness to change*) (Schwartz, 2012). Such self-restriction and resistance to change is highly compatible with religiosity on a theoretical and empirical level, as prior research has demonstrated (Roccas and Elster, 2013, p. 195). The second set of values we are interested in are those that emphasize the well-being of other people (*self-transcendence*), which contrast others that reflect the prioritization of one's own interests (*self-enhancement*) (Schwartz, 2012). As we elaborate below, the connection between *self-transcendence* and religiosity are more complex. Finally, because it directly opposes one of the core functions of religion (see below), we also examine differences in the *hedonism* value, which belongs to both higher-order values *self-enhancement* and *openness to change* (Schwartz, 2012).^1^

Generally, religiosity is strongly connected to the values of *conservation* because these values have a strong focus on tradition. This is very compatible with religion's “central goals of submission to transcendental authority and protecting individuals from uncertainty” (Roccas and Elster, 2013, p. 195). These theoretical considerations are backed by evidence collected in the meta-analysis of Saroglou et al. (2004), who show that religious people tend to favor values that belong to the higher-order dimension of *conservation*, especially its sub-values *tradition*/*conformity* (and tend to dislike values of *self-enhancement* or *openness to change*). The higher-order dimension of *conservation* is, thus, particularly relevant when comparing religious with non-religious groups. This leads to our first hypothesis:

- Hypothesis 1: Muslim immigrants (and Christian natives) score higher in *conservation* values (*tradition*/*conformity* and *security*) (*Conservation Hypothesis*).

In contrast to *conservation* related values, the *hedonism* value is highly incompatible with religion. This is because the *hedonism* value expresses the goal of having fun and enjoying oneself to the most (Schwartz, 2012). The gratification of material desires directly opposes one of the primary principles of religion, which is to “temper self-indulgent tendencies” (Roccas and Elster, 2013, p. 195). For Christians as well as Muslims, this pattern is also confirmed by the meta-analysis of Saroglou et al. (2004). Thus, we hypothesize that:

- Hypothesis 2: Muslim immigrants (and Christian natives) score lower in *hedonism* compared to non-religious people (*Hedonism Hypothesis*).

The connection between the higher-order value *self-transcendence* and religiosity is more ambiguous. According to Schwartz (2012), *self-transcendence* consists of the two values *universalism* and *benevolence*. What these values have in common is that they measure compassion with and concern for other people. However, what separates *universalism* and *benevolence* is the scope of these concerns. While *benevolence* refers to people who are close to oneself, *universalism* applies to all people, which should also include members of, for example, other religious groups (Roccas and Elster, 2013, p. 195). *Universalism* is, thus, an eminently important value when it comes to the evaluation of different ethnic or religious out-groups (Eisentraut, 2019) and expresses the goal of appreciation and tolerance. Most religions, including Christianity and Islam, emphasize selflessness with close others. Hence, *benevolence* is compatible with religiosity (Roccas and Elster, 2013, p. 195), which is also found in most empirical studies (Saroglou et al., 2004). In contrast, however, religious people tend to score low on *universalism*, especially in Mediterranean countries (Saroglou et al., 2004). This can be explained by the particularisms of religions that make *universalism* less compatible with religiosity (Roccas and Elster, 2013, p. 195). Based on these considerations, we formulate the two hypotheses that:

- Hypothesis 3a: Muslim immigrants (and Christian natives) score higher in *benevolence* compared to non-religious people (*Benevolence Hypothesis*)
- Hypothesis 3b: Muslim immigrants (and Christian natives) score lower in *universalism* compared to non-religious people (*Religious Universalism Hypothesis*).

Our argumentation thus far mainly differentiates between religious and non-religious individuals. That is, hypotheses 1 to 3 implicitly contain that value differences between Christian natives and Muslim immigrants are negligible. On the one hand, this reasoning is in line with the meta-analysis of Saroglou et al. (2004), who report, on average, rather similar value patterns across Christians and Muslims in previous research. This is mirrored by that fact that European Christians do not seem to discriminate Muslims more than non-religious people do (Czymara and Schmidt-Catran, 2016, p. 214). Helbling and Traunmüller (2018) conclude that the “current political conflict is not about Muslims vs. Christians or immigrants vs. natives, but about political liberalism vs. religious fundamentalism” (p. 15). Similarly, van der Noll and Saroglou (2015) find that Christians rather support Islamic education than abolishing religious education altogether. However, the pure focus on the role of religiosity neglects potential differences also between different branches of religion. Moreover, it does not take into account the specific situation of most European Muslims, that is, their status as a religious, and often ethnic^2^, minority within each of the host societies we analyze. Hence, there are reasons to assume differences also within the category “religious,” depending on the particular denomination or ethnic minority status that make it worthwhile to test potential differences between these groups, as we will elaborate in the following.

Previous research suggests that Muslims in Europe tend to exhibit higher values of traditionalism (Connor, 2010) and fundamentalism (Koopmans, 2015) compared to Christians, which translates into, for example, dismissive attitudes toward homosexuality (van Den Akker et al., 2013; Jäckle and Wenzelburger, 2015) or gender equality (Diehl et al., 2009). While neither traditionalism nor fundamentalism are identical to the basic human value *conservation*, there is conceptual overlap, especially with its sub-values *tradition* and *conformity*. In the context of Islam, *tradition* relates to “eternal” rules that are binding for its believers (Koopmans, 2015) and a literalist reading of the Quran. Submission, the literal translation of *Islam*, to God and the collective belonging to one *Ummah* (Tibi, 2010) can be understood as a form of religious *conformism* that is especially pronounced in Islam. In the European context, such religious conformism among Muslims might be boosted by religious gatherings with conservative peers, as prior research indicates that mosque attendance predicts support for patriarchal values in non-Muslim societies (Alexander and Welzel, 2011). Hence, while *conservation* should generally be larger for religious compared to non-religious individuals, as we elaborated above, this value might be even more prevalent in Muslims compared to Christians in Europe due to higher levels of traditionalism/fundamentalism. Moreover, traditionalism or fundamentalism might be seen as a form of religiosity. In this case, differences in *conservation* should be explained by different levels of religiosity between Muslims and Christians (Simsek et al., 2019). On the other hand, a literalist reading and collective belonging might make religiosity a stronger predictor of *conservation* for Muslims than for Christians. While we will test these considerations, for now, we formulate the following hypothesis:

- Hypothesis 4: Muslims exhibit higher levels of *conservation* than Christians do (*Muslim Conservation Hypothesis*).

On the other hand, native Europeans' tend to view Muslim immigrants particularly negatively (Strabac and Listhaug, 2008; Bansak et al., 2016; Czymara and Schmidt-Catran, 2017; Czymara, 2019), in some incidents even including violence and hate crimes targeted against Muslims (Borell, 2015). Moreover, the rhetoric of political elites, especially on the far-right, on Muslims is often very hostile (Peachey, 2018), and links Muslim immigration primarily to Islamic terrorism (Kaminski, 2015; Waterfield, 2015). (Potentially) being the target of hate speech and violence could be another reason why Muslims might score higher in the *security* dimension of the *conservation* value (see hypothesis 1). Furthermore, experiences of discrimination may increase sensitivity toward social exclusion. This, in turn, may boost values of tolerance and equal opportunities. This is what the human value *universalism* captures. The particular social position of Muslims in Europe might, hence lead to the special situation where religion is not only associated with particularistic compassion toward those who are close, but a universal one toward people in general. In this case, being Muslim should not only be related to scoring higher on self-transcendence's *benevolence* sub-value (see hypothesis 3a) but also on its *universalism* value. This leads to our final hypothesis, which partly competes with hypothesis 3b:

- Hypothesis 5: Muslims in Europe exhibit higher levels of *universalism* than Christians do (*Discrimination Universalism Hypothesis*).

## Muslim Immigration in Germany, France, Belgium, and Sweden

From a global perspective, many European countries, including those destinations that are part of the present analysis, can be regarded as rather exceptional terms of culture and values. Schulz et al. (2019) describe European countries as “Western, Educated, Industrialized, Rich, and Democratic” (WEIRD) and argue that higher levels of individualism, liberalism, and social trust are rooted in a historical decrease of kin-based institutions caused by rules of the Western Church. In contrast, many sending countries, including Muslim ones, are characterized by high(er) kindship intensity and collectivism (Tibi, 1994), which should then lead to value differences between (descendants of) Muslim immigrants and non-Muslim Europeans.

The four European countries we analyze are all popular destinations for immigrants—including a sizable, and growing, Muslim population (Koopmans, 2013; Pew Research Center, 2017), a point that is crucial for our study. In all countries, Muslims are considerably younger than the average population (De Raedt, 2004; also see Table 1) and tend to have higher fertility rates, leading to a predicted increase in their population share even without any future immigration (Pew Research Center, 2017). Political elites as well as the general public often vividly debate the inflow and demographics of Muslims in Europe (Czymara, 2019). Yet, all four countries have distinct immigration histories, different economies, and integration politics. In the following, we will give a brief description of the situation of Muslims in each country.

**Table 1:** Number of cases and demographics.

	**Belgium (N)**	**Germany (N)**	**France (N)**	**Sweden (N)**	**Religiosity [mean (SD)]**	**Gender (percentage male)**	**Age [mean (SD)]**	**Education—ISCED (median)**
Muslim immigrants	584	508	426	268	7.5 (2.4)	54.1	34.6 (13.4)	3: Upper Secondary, lower tier (17.7%)
Christians natives	4,483	10,145	4,155	3,418	5.8 (2.3)	44.8	53.6 (18.8)	3: Upper Secondary, lower tier (30.6%)
Non-religious natives	6,693	8,837	6,164	7,993	2.3 (2.5)	53.0	45.5 (18.0)	4: Upper secondary, upper tier (16.0%)
Total	11,760	19,490	10,745	11,679	3.9 (3.0)	49.7	48.5 (18.8)	3: Upper Secondary, lower tier (28.4%)

*Data source: European Social Survey waves one to eight*.

*Belgium* can be considered “*one of the most multicultural and multiracial countries of the European Union“* (Martiniello, 2020, p. 225). While Belgium has a rather liberal integration policy, it lies in the midfield regarding religious rights for Muslims (Koopmans, 2013) and regarding public sentiments toward Muslim immigrants (Strabac and Listhaug, 2008; Savelkoul et al., 2012; Czymara, 2019). Most of Belgium's Muslim population consists of immigrants from Morocco and Turkey, and their descendants (De Raedt, 2004; Koopmans, 2015). In 1974, Belgium was the first European country to recognize Islam as an official religion and from the mid-1980 on, Islam was increasingly present in the Belgian public (De Raedt, 2004). The history and tradition of ethnic and religious diversity might make it more likely that Muslim immigrants in Belgium and Christian as well as non-religious native Belgians hold values that are more similar compared to countries with less history of diversity^3^.

Most Muslims in *France* originate from the Maghreb, due to France's history of holding colonies in this area. Immigration laws gave many Muslims from former colonies the possibility to gain French citizenship (Croucher, 2013). However, France's strong tradition of *laïcité*, the strict separation of church and state, leads to policies that are rather restrictive for all religions, including Islam. For example, there is no religious education in schools, no confessional schools and neither teachers nor students are allowed to wear a headscarf (or any other religious symbols) at school or any other public institution (Koopmans, 2013; Kuru, 2016). This leads to tensions between secularists and religious groups in general, and Muslims in particular. In addition, religious fundamentalism seems to be particularly widespread among Muslims in France (Koopmans, 2015). While Frenchmen are indeed most likely to reject the religious headscarf (Helbling, 2014), public opinion toward Muslim immigrants does not seem to be particularly negative, relatively to other countries in Europe (Strabac and Listhaug, 2008; Savelkoul et al., 2012; Helbling, 2014; Czymara, 2019). In recent years, several Islamist terror attacks of Islamists shocked France (see, for example, Jungkunz et al., 2018; Vasilopoulos et al., 2018). On the one hand, France's long history of diversity may have caused a convergence of values between Muslim immigrants, Christian and non-religious natives. We might, thus, expect that human values of Muslim immigrants, Christian and non-religious natives are relatively similar in France. On the other hand, strict secularism may have led to clashes between religious groups and non-religious native Frenchmen. Hence, one could also hold the competing expectation that human values of religious groups (Muslim immigrants and Christian natives) differ more strongly from those of non-religious natives.

*Germany* began to receive sizeable numbers of Muslims from Turkey from the 1960s on, based on a treaty between the German and the Turkish governments. These migrants were recruited as *Gastarbeiter* (“guest workers”) and meant to contribute to Germany's labor market, primarily filling temporary labor shortages (Ellermann, 2015). Contrary to the initial plan, large-scale immigration and family unification lead to “*unanticipated and, ultimately, unwanted mass immigration”* for Germany (Ellermann, 2015, p. 1236). This may explain why Germany grants relatively few religious rights to Muslims, ranking just above France in this respect (Koopmans, 2013). However, German Muslims seem to be less religiously fundamentalist compared to their counterparts in other European countries (Koopmans, 2015) and the German public is relatively neutral (Strabac and Listhaug, 2008; Helbling, 2014) or even positive (Czymara, 2019) toward Muslim immigrants (but see Savelkoul et al., 2012). During the refugee flows to Europe in 2015/16, Germany received most refugees in total, who primarily originated from Muslim areas (Pew Research Center, 2017). The intake of a large number of refugees was connected to some dramatic events. Some acts were committed by refugees such as the sexual assaults taking place on New Year's Eve 2015/16 (Czymara and Schmidt-Catran, 2017) or the Islamist terror attack on a Christmas market in Berlin in 2017 (Fischer-Preßler et al., 2019; Schmidt-Catran and Czymara, 2020). Other acts were committed by German natives, such as personal attacks against refugees or arson attacks on asylum shelters (Jäckle and König, 2018). Historically relatively reluctant integration politics and recent inter-ethnic tensions might lead to expect larger value differences in Germany.

Finally, *Sweden* has the youngest history of Muslim immigration, with Muslims being largely absent in the Swedish population before the 1980s, but with a steady increase afterwards (Bevelander and Otterbeck, 2010). Sweden's Muslims tend to exhibit rather low levels of religious fundamentalism (Koopmans, 2015) and Swedish natives are consistently the most Muslim friendly in Europe (Strabac and Listhaug, 2008; Helbling, 2014; Czymara, 2019). In contrast to Germany, only few of Sweden's Muslims entered the country as guest workers, while most were refugees or their families (Bevelander and Otterbeck, 2010). This tendency further rose in 2015, when Sweden accepted a relatively large number of refugees (Pew Research Center, 2017). Drawing upon the reasoning of Schulz et al. (2019), Sweden is an especially “WEIRD” country, with a strong welfare state and a long tradition of cultural liberalism and social democracy. Differences in values between Muslim immigrants and native non-Muslim Swedes might thus be especially strong. Moreover, Sweden has a very multi-culturalist approach to diversity, aimed at preserving origin cultures instead of assimilation. Therefore, Sweden grants most religious rights to Muslims from all countries analyzed, while having little requirements for civic integration (Koopmans, 2013). Pre-existing value gaps in combination with Sweden's multi-cultural immigration politics (Koopmans, 2013), might lead to lower investments of Muslim immigrants in cultural and social capital (Esser, 2010) and, hence, less assimilation and the persistance of value differences (Kymlicka, 1995). Given these considerations, we expect that value differences between Muslim immigrants and (Christian and non-religious) native Swedes are most pronounced compared to the other three countries of investigation.

## Data

We draw upon pooled data from the first eight waves of the European Social Survey (European Social Survey Cumulative File ESS, 2018) to examine how basic human values distribute across Muslim immigrants and Christian natives in Europe. To capture basic human values, the ESS uses the Portrait Value Questionnaire (PVQ) 21, a modification of the PVQ-40 (Schwartz, 2007). It describes portraits of different people and asks respondents to evaluate how similar this portrait is to them. Each of the five values were measured by two items: *universalism* by the importance of equality and understanding other people, *benevolence* by the importance of the well-being of others and loyalty toward friends, *conformity* by the importance of following rules and behaving properly, *tradition* by the importance of being modest and following customs and religion, *security* by the importance of living in secure surroundings and having a strong state that defends its citizens and *hedonism* by the importance of having a good time and to do things that give pleasure. Responses ranged from 1 (very much like me) to 6 (not like me at all) and were recoded so that high scores indicated a high importance of the value. See Table 3 for the wording of the items measuring each human value.

To differentiate the religious denominations, we use the following ESS item: “*Do you consider yourself as belonging to any particular religion or denomination*?” Using the responses “*Islamic”* for Muslims and combining “*Roman Catholic,” “Protestant,” “Eastern Orthodox,”* and “*Other Christian denomination*” for Christians. We define non-religious individuals as those stating that they do not belong to a religion or denomination at present. To account for religiosity, we draw upon the item “*Regardless of whether you belong to a particular religion, how religious would you say you are?”* with answers ranging from 0 (“*Not at all religious*”) to 10 (“*Very religious*”). Importantly, this question is asked for all respondents, including “believers without belonging” (i.e., those that are religious but not part of an official denomination)^4^.

In this study, we understand immigrants as first and second generation immigrants (i.e., people with a migration background), while we refer to those without a migration background as natives. We define a migration background as an individual who is herself not born in the country of residence or whose mother or father was not born in this country^5^.

For deeper analysis, we account for differences in socio-economic status (SES), which we here capture with age and education. We measure a person's age in years. For education, we use the *International Standard Classification of Education* (ISCED). Table 1 gives an overview of the descriptive statistics of these variables for each of the three groups we want to compare in each county. As Table 1 shows, Muslim immigrants are clearly a minority in each country, their total numbers range from 268 in Sweden to 584 in Germany (which is sufficient for our SEM approach). Furthermore, Sweden is the least religious country of our comparison, with almost 70 percent of our sample of analysis being non-religious natives. In contrast, with more than half of the analyzed sample, Germany has the highest amount of Christian natives.

## Method

Similar to previous studies (i.e., Davidov et al., 2019) we employ multi-group confirmatory factor analysis (MGCFA), to analyze the differences in the values *universalism, benevolence, tradition, conformity, security* and *hedonism* of Muslim immigrants, Christian natives and non-religious natives^6^. MGCFA is a method that allows to test whether a hypothesized measurement model fits with the data that are used (Jöreskog, 1977). In our case, this means that we can test if the ESS human value scale items really measure their respective values (as latent constructs) and if the same constructs are measured in every group.

Because our main aim is to analyze statistical differences for values between the three (non-)religious groups in each country, we estimate four separate models—one for each country. The studies of Davidov (2008, 2010) show that when using the human value scale of the ESS, only a subset of countries share the same value structure. For Belgium, Germany, France and Sweden the same value structure can be found empirically, which enables us to use four identical models that are comparable (Davidov, 2008). Those studies also show that only a certain amount of values can be used at the same time when using confirmatory factor analysis with the ESS human value scale. Beside our theoretical considerations, this is one of the reasons to not include more human values into the analysis—the statistical model would simply not work anymore (for a deeper analysis of the statistical difficulties when working with the ESS human value scale, see Davidov, 2008, 2010).

For the statistical comparison of the value means between the (non-)religious groups, we need to make sure that the understanding of the ESS items is the same between those groups. To empirically test this, we have to check for measurement invariance, which is a statistical method to ensure comparability of construct measurements between groups or points in time (Milfont and Fischer, 2010). A prerequisite for meaningful comparisons of latent means (in our case, values) between groups is scalar invariance. Scalar invariance means that the factor loadings of the constructs are invariant across groups (which would be metric invariance) and the intercepts of the indicators are invariant as well (Vandenberg and Lance, 2000). Without at least partial scalar invariance, it would not be possible to compare the different religious groups. This is because, in this case, we would measure different constructs/values in the different groups (Milfont and Fischer, 2010). Our analysis reveals that, fortunately, we establish partial scalar invariance across groups. The only item that is not invariant is *imptrad (“Tradition is important to him/her. He/she tries to follow the customs handed down by his/her religion or his/her family)*. This item has to be estimated freely for non-religious natives, which is no surprise due to its item formulation. After all, “religion” is directly adressed in the question wording and therefore this item surely does not measure the same when asking religious vs. non-religious individuals. Following the criteria of Chen (2007), all other items are scalar invariant as can be seen in the model fit for different invariance levels in Table 2. Therefore, we can compare the latent means between the different (non-)religious groups.

**Table 2:** Fit measures for the different levels of measurement invariance.

**Country**	**Global fit measures**	**Configural**	**Metric**	**Partial scalar**	**Scalar**
Belgium	Pclose RMSEA CFI SRMR	0.994 0.046 0.953 0.027	1.000 0.044 0.953 0.028	1.000 0.044 0.948 0.030	0.001 0.054 0.919 0.043
France	Pclose RMSEA CFI SRMR	0.980 0.044 0.959 0.028	1.000 0.044 0.958 0.030	1.000 0.045 0.954 0.032	0.000 0.065 0.901 0.052
Germany	Pclose RMSEA CFI SRMR	0.986 0.047 0.958 0.031	1.000 0.045 0.958 0.032	1.000 0.044 0.955 0.032	00.00 0.057 0.926 0.042
Sweden	Pclose RMSEA CFI SRMR	0.410 0.050 0.953 0.033	0.906 0.048 0.953 0.035	0.909 0.048 0.948 0.036	0.000 0.057 0.927 0.044

*Global fit measures (cut-off criteria in brackets)*.

*Pclose = probability of close fit (≥0.05)*.

*RMSEA = root mean square error of approximation (<0.06)*.

*CFI = comparative fit index (>0.95)*.

*SRMR = standardized root mean square residual (<0.08)*.

*See West et al. (2012) for details on fit measures*.

**Figure 2:**
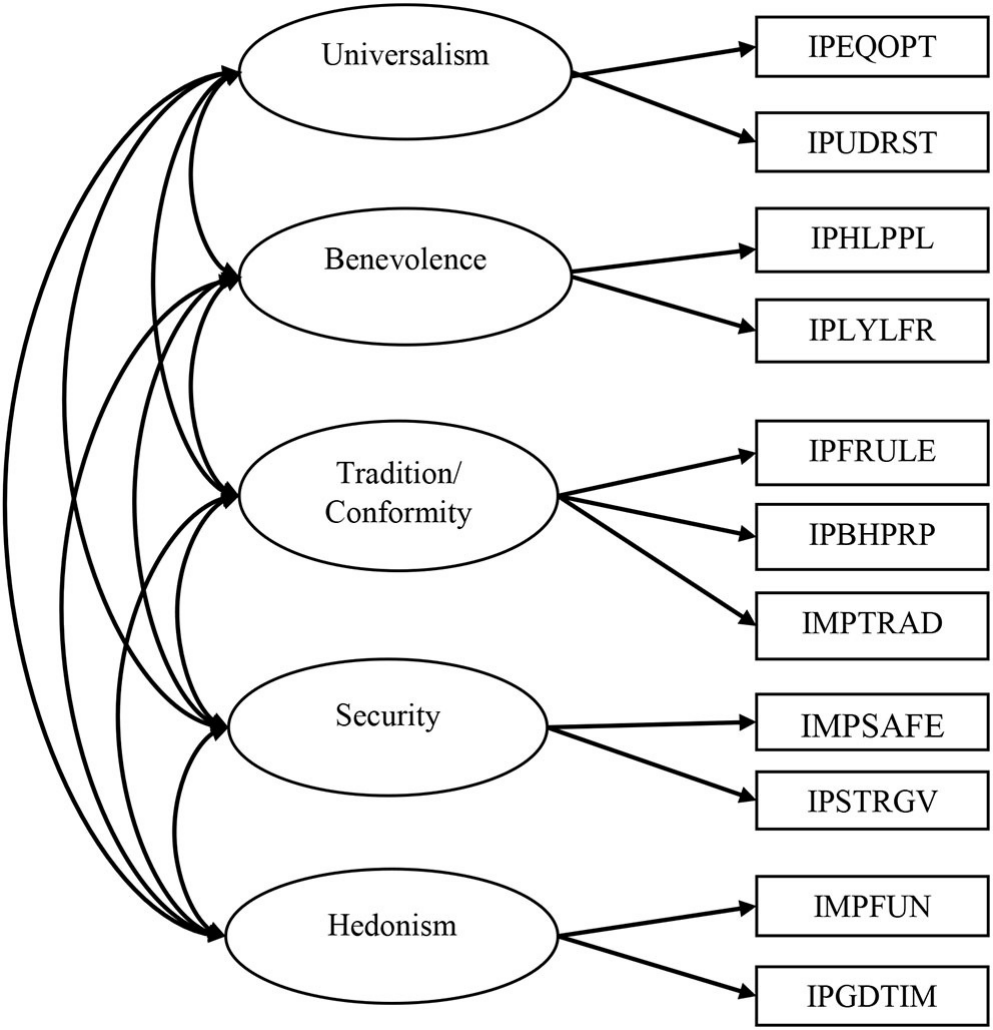
Multi-group confirmatory factor analysis.

As the only modification to our theoretically assumed model, we had to build one value that consists of the values *tradition* and *conformity* (similar to Davidov et al., 2019). One item of the *tradition/conformity* construct had to be excluded from the analysis due to cross-loadings. Figure 2 shows our final model.

Our main interest lies in describing the total differences in human values between Muslim immigrants compared to Christian natives as well as non-religious natives. However, we also run two additional models to account for differences in religiosity and socio-economic status between the three groups. That is, we control for the effects that religiosity and socio-economic status (age and education) have on each value in each country for each group. These “control” models are rather demanding for our data since they imply a plethora of additional parameters (one for each control variable on each value for each group within each country). This results in an exponential growth of the complexity of the models with each additional control variable. Hence, while these models can offer interesting insights, we want to emphasize that some fit metrics of these models do not meet the threshold that is usually seen as reliable (see below).

## Results

### Descriptive Overview

Table 3 provides the means values for all our items in each of the four countries. This descriptive evidence already suggests that both religious groups, Muslim immigrants and Christian natives, agree stronger to the *conservation* related items *conformity, tradition*, and *security* compared to non-religious natives in all four countries, lending preliminary support for the *Conservation Hypothesis*. Contrary to our expectations, the same is also true for the *universalism* items, where the non-religious actually score lowest. This contradicts our *Universalism Hypothesis* (H3b), but is in line with the *Muslim Universalism Hypothesis* (H5). Differences are smaller but mostly in the expected direction for the *benevolence* items, lending some preliminary support to the *Benevolence Hypothesis*. Regarding *hedonism*, the picture is not clear. Christians indeed tend to disagree most to the *hedonism* items, which would support the *Hedonism Hypothesis*. Interestingly, however, Muslims hold similar scores in these items to non-religious natives, which is not in line with the *Hedonism Hypothesis*. We turn to our SEM models to test these differences more thoroughly.

**Table 3:** Means for the value measuring items for Belgium, France, Germany, and Sweden.

**Value**	**Item name**	**Question wording (response categories: 1 not like me at all−6 very much like me)**	**Item means for Belgium (BE), France (FR), Germany (GE), and Sweden (SW)**			
		**Here we briefly describe some people. Please read each description and think about how much each person is or is not like you. Tick the box to the right that shows how much the person in the description is like you**		**Muslims**	**Christians**	**Non-religious**
Universalism	Ipeqopt	He/she thinks it is important that every person in the world be treated equally. He/she believes everyone should have equal opportunities in life	BE	5.14	4.97	4.93
			FR	5.35	5.07	5.17
			GE	5.17	4.92	4.92
			SW	5.53	4.99	4.95
	Ipudrst	It is important to him/her to listen to people who are different from him/her. Even when he/she disagrees with them, he/she still wants to understand them	BE	4.79	4.69	4.65
			FR	4.90	4.62	4.68
			GE	4.83	4.82	4.79
			SW	5.13	4.55	4.48
Benevolence	Iphlppl	It's very important to him/her to help the people around him/her. He/she wants to care for their well-being	BE	5.04	4.96	4.86
			FR	5.00	4.66	4.57
			GE	4.99	4.91	4.85
			SW	5.24	4.76	4.68
	Iplylfr	It is important to him/her to be loyal to his/her friends. He/she wants to devote herself to people close to him/her	BE	5.16	5.26	5.22
			FR	5.15	5.10	5.07
			GE	5.24	5.27	5.28
			SW	5.40	5.06	5.03
Conformity/ Tradition	Ipfrule	He/she believes that people should do what they're told. He/she thinks people should follow rules at all times, even when no-one is watching	BE	4.23	4.03	3.61
			FR	3.44	3.26	2.94
			GE	3.98	3.66	3.45
			SW	4.31	3.80	3.55
	Ipbhprp	It is important to him/her always to behave properly. He/she wants to avoid doing anything people would say is wrong	BE	4.69	4.64	4.32
			FR	4.50	4.45	4.16
			GE	4.51	4.16	3.97
			SW	4.43	3.96	3.79
	Imptrad	Tradition is important to him/her. He/she tries to follow the customs handed down by his/her religion or his/her family	BE	5.08	4.72	3.92
			FR	4.80	4.25	3.03
			GE	4.94	4.41	3.66
			SW	4.75	4.35	3.64
Security	Impsafe	It is important to him/her to live in secure surroundings. He/she avoids anything that might endanger his/her safety	BE	4.86	4.64	4.39
			FR	4.75	4.40	4.14
			GE	4.81	4.60	4.49
			SW	4.77	3.98	3.82
	Ipstrgv	It is important to him/her that the government insure his/her safety against all threats. He/she wants the state to be strong so it can defend its citizens	BE	4.70	4.56	4.37
			FR	4.77	4.61	4.23
			GE	4.84	4.65	4.62
			SW	5.03	3.95	3.89
Hedonism	Impfun	Having a good time is important to him/her. He/she likes to “spoil” him/herself	BE	4.33	4.30	4.51
			FR	3.92	3.67	3.98
			GE	3.87	3.73	3.98
			SW	4.73	4.20	4.30
	Ipgdtim	He/she seeks every chance he/she can to have fun. It is important to him/her to do things that give him/her pleasure	BE	4.40	4.25	4.48
			FR	4.71	4.50	4.72
			GE	4.47	4.37	4.58
			SW	3.71	4.00	4.02

### Value Differences Between Groups

Generally, the results of the MGCFA are in line with the *Conservation Hypothesis* that Muslims and Christians score higher in the conservation values (*tradition*/*conformity* and *security)* compared to non-religious natives as Table 4 shows. Non-religious people are clearly least associated with the *conservation* value, which is true throughout all four countries. However, there is also considerable difference between the two religious groups. Islam, in this respect, outperforms Christianity in encouraging *traditionalist* and *conformist* values, which is in line with prior research showing higher levels of traditionalism among Muslims compared to Christians in Europe (Connor, 2010; Koopmans, 2015). Second, Muslims seem to value a strong state, as indicated by the high level of agreement with *security*, which, besides being related to conservation of social and individual order, might also be the result of potentially living under threat in various European contexts. We, thus, also find support for our *Muslim Conservation Hypothesis*. While the reported differences are statistically significant in all four countries, the high level of agreement to the *conservation* values among Muslims is particularly true for Sweden and somewhat less for France and Belgium.

**Table 4:** Comparison of latent means of values.

	**Value**	**Muslims compared to Christians**	**Muslims compared to non-religious**
Belgium	Universalism Benevolence Tradition/Conf. Security Hedonism	+0.113^***^ +0.026 +0.106^**^ +0.183^***^ +0.094^*^	+0.156^***^ +0.109^**^ +0.448^***^ +0.387^***^ −0.102^*^
France	Universalism Benevolence Tradition/Conf. Security Hedonism	+0.222^***^ +0.226^***^ +0.079 +0.223^***^ +0.202^***^	+0.164^***^ +0.286^***^ +0.318^***^ +0.540^***^ −0.021
Germany	Universalism Benevolence Tradition/Conf. Security Hedonism	+0.107^**^ +0.028 +0.326^***^ +0.201^***^ +0.102^*^	+0.126^**^ +0.056 +0.520^***^ +0.275^***^ −0.087
Sweden	Universalism Benevolence Tradition/Conf. Security Hedonism	+0.495^***^ +0.595^***^ +0.490^***^ +0.932^***^ +0.406^***^	+0.544^***^ +0.656^***^ +0.701^***^ +1.040^***^ +0.273^**^

* *p < 0.05*;

** *p < 0.01*;

*** *p < 0.001*.

Accounting for the different levels of religiosity, differences between Muslims and Christians in *tradition*/*conformity* remain statistically significant only in Sweden, where the effect parameter actually switches its direction (see Table 5). There is no clear pattern regarding security once we control for religiosity. Hence, the idea that the degree of religiosity mediates the association between religion and values only finds limited support in our model. However, one should treat the results of Tables 5, **7** with some caution, as the model is somewhat demanding for our data basis^7^(for more information on fit measures and cut-off criteria, see Table 2). What we do find, however, is that religiosity is consistently positively associated with higher levels of *tradition/conformity* as Table 6, which is based on the same model, shows^8^. This is true for all countries and all groups (even those not currently belonging to a religious denomination). Hence, religiosity has a stronger impact on *tradition/conformity* for some groups than for others. However, the size of this association is not consistent throughout countries: While religiosity has a slightly stronger effect on *tradition/conformity* for Christians in Belgium and France, its effect is much stronger for Muslims in Germany and Sweden. In contrast to religiosity, Table 7^9^shows that the group differences regarding *tradition/conformity* stay rather robust when controlling for age and education, with the exception of Sweden. Noticeably, this is true although age and education are correlated with *tradition/conformity* (age positively, education negatively).

**Table 5:** Comparisons of latent means of values when controlling for religiosity.

	**Value**	**Muslims compared to Christians**	**Muslims compared to non-religious**
Belgium	Universalism Benevolence Tradition/Conf. Security Hedonism	+0.108 +0.039 +0.081 +0.462^**^ +0.026	−0.039 −0.103 +0.003 +0.328^**^ +0.028
France	Universalism Benevolence Tradition/Conf. Security Hedonism	+0.435^***^ +0.609^***^ +0.109 +0.371^*^ +0.314	+0.272^*^ +0.444^**^ +0.052 +0.477^**^ +0.168
Germany	Universalism Benevolence Tradition/Conf. Security Hedonism	+0.417^***^ +0.034 +0.116 +0.105 −0.100	+0.297^**^ −0.133 −0.058 +0.043 −0.109
Sweden	Universalism Benevolence Tradition/Conf. Security Hedonism	+0.281^**^ +0.587^***^ −0.265^***^ +0.480^**^ +0.924^***^	+0.172 +0.469^***^ −0.164 +0.609^***^ +0.767^***^

* *p < 0.05*;

** *p < 0.01*;

*** *p < 0.001*.

**Table 6:** Standardized effects of religiosity on values.

	**Value**	**Muslims**	**Christians**	**Non-religious**
Belgium	Universalism Benevolence Tradition/Conf. Security Hedonism	+0.103 +0.102^*^ +0.298^***^ +0.054 −0.048	+0.139^***^ +0.161^***^ +0.314^***^ +0.202^***^ −0.100^***^	+0.042^*^ +0.108^***^ +0.203^***^ +0.113^***^ −0.034^*^
France	Universalism Benevolence Tradition/Conf. Security Hedonism	−0.056 −0.050 +0.198^**^ +0.056 −0.079	+0.097^***^ +0.148^***^ +0.284^***^ +0.170^***^ −0.058^**^	+0.029 +0.053^**^ +0.181^***^ +0.126^***^ −0.052^**^
Germany	Universalism Benevolence Tradition/Conf. Security Hedonism	−0.095 +0.120 +0.307^***^ +0.081 +0.003	+0.139^***^ +0.151^***^ +0.172^***^ +0.044^**^ −0.091^***^	+0.078^***^ +0.041^**^ −0.055^***^ −0.092^***^ −0.016
Sweden	Universalism Benevolence Tradition/Conf. Security Hedonism	+0.427^***^ +0.203^*^ +0.462^***^ +0.294^**^ −0.327^**^	+0.164^***^ +0.147^***^ +0.164^***^ +0.025 −0.016	+0.004 +0.019 +0.152^***^ +0.077^***^ −0.054^***^

* *p < 0.05*;

** *p < 0.01*;

*** *p < 0.001*.

**Table 7:** Comparisons of latent means of values when controlling for age and education.

	**Value**	**Muslims compared to Christians**	**Muslims compared to non–religious**
Belgium	Universalism Benevolence Tradition/Conf. Security Hedonism	+0.292^**^ −0.051 +0.431^***^ −0.262^*^ −0.089	+0.333^**^ −0.025 +0.719^***^ −0.075 −0.200
France	Universalism Benevolence Tradition/Conf. Security Hedonism	+0.340^*^ −0.228 +0.317 −0.399 −0.320	+0.439^**^ −0.140 +0.352^*^ −0.201 −0.050
Germany	Universalism Benevolence Tradition/Conf. Security Hedonism	+0.165 −0.237^*^ +0.357^**^ −0.204 +0.007	+0.294^**^ −0.108 +0.597^***^ +0.051 −0.095
Sweden	Universalism Benevolence Tradition/Conf. Security Hedonism	+0.829^***^ +0.407^*^ +0.161 +0.663^**^ +0.091	+0.977^***^ +0.550^**^ +0.187 +0.614^**^ −0.004

* *p < 0.05*;

** *p < 0.01*;

*** *p < 0.001*.

*Security*, on the other hand, only positively correlates with religiosity for Christians in all four countries. For Muslims, there is no connection between religiosity and *security*, except for the case of Sweden. More importantly for our study, the higher levels of *security* among Muslim immigrants do not seem to be strongly connected to religiosity. Rather, group differences in *security* values are due to differences in socio-economic status, as controlling for socio-economic status renders most group differences in *security* statistically insignificant (see Table 7). This finding is not surprising as *security* has a strong positive correlation with age as well as a negative correlation with education.

There is only little empirical support for lower levels of *hedonism* among Muslims. In fact, we only find that Muslims are statistically significantly less hedonistic compared to non-religious people in Belgium. In contrast to our theoretical expectations, Muslims are even more hedonistic than the non-religious in Sweden. In the case of Germany and France, there are no significant differences between Muslims and non-religious individuals regarding *hedonism*. Moreover, our results suggest that Muslims are consistently more hedonistic compared to Christians in all four countries. However, these somewhat surprising findings can be understood by that fact that Muslims are significantly younger than the other two groups (see Table 1). Younger people are usually more hedonistic than older individuals are. Hence, accounting for differences in the age structure and education, all differences between Muslims and the two other groups are no longer statistically different from zero as Table 7 shows.

Regarding *self-transcendence*, we expected that religious people, i.e., Muslim immigrants and Christian natives, agree more to the *benevolence* items but less to *universalism* items. However, as Table 4 shows, Muslims actually score higher in both *benevolence* and *universalism*—and this is true compared to both Christians and non-religious people. The demarcation line for values related to *universalism*, thus, seems not to separate the religious and the non-religious, but rather immigrants and natives. While this is consistent with the *Benevolence Hypothesis*, it is in direct contrast with the *Religious Universalism Hypothesis* and rather supports our *Discrimination Universalism Hypothesis*. Above we show that Muslim immigrants in Europe tend to be more traditional. Yet, they are simultaneously in favor of equal opportunities for all. While this reasoning has to remain speculative at this point, this might stem from own experiences of discrimination coming from being a religious minority in Europe. At least, higher levels of *universalism* among Muslims can more clearly be explained by discrimination experiences than by religion, as religious people usually score low in *universalism* (Saroglou et al., 2004; Roccas and Elster, 2013). The higher agreement of Muslims to *universalism* compared to the other two groups is statistically significant in all four countries but, again, most pronounced in Sweden. With the exception of Belgium, this even holds when controlling for religiosity, as Table 5 shows. The differences regarding *universalism* between the three groups are robust when controlling for socio-economic status even though there is a significant positive correlation between education and *universalism*.

The cross-national differences we find are largely in line with the idea that, first, a national tradition of diversity and experience with immigration (as in France and Belgium) is connected to fewer differences in values between groups. A longer tradition of immigration and cohabitation, hence, seems to facilitate the social and normative integration of immigrants. In contrast, differences are usually most pronounced in Sweden, where Muslim presence is a relatively new phenomenon (Bevelander and Otterbeck, 2010). However, the similar results for Germany might be somewhat surprising in this respect, since Germany long hesitated with being ethnically diverse (Ellermann, 2015). This might be explained by national differences in integration politics. Stricter politics, as they are implemented in Germany, are meant to incentivize assimilation and might, thus, lead to a convergence of values between minority groups and the native majority. Hence, a possible explanation is that Muslim immigrants in such contexts invest more into the social and cultural capital (Esser, 2010). Similarly, differences are smaller in France, where there is a relatively rigorous legal separation of church and state (Koopmans, 2013). Notably, France's *laïcité* did not result in larger value differences between religious and non-religious groups compared to the other countries. In contrast, value differences are strongest in Sweden, which has a multi-cultural integration policy, is generous regarding religious rights (Koopmans, 2013), and has a public that is characterized by relatively high levels of tolerance (Strabac and Listhaug, 2008; Helbling, 2014; Czymara, 2019), which might translate into fewer investments into social and cultural capital of immigrants (Esser, 2010). However, Belgium has a multi-cultural approach to immigrant integration as well (Koopmans, 2013), but shows significantly smaller differences between Muslim immigrants and natives compared to Sweden. Hence, an interplay between historical aspects and current politics might be the key when explaining existing value differences across countries.

## Conclusion

Native (non-Muslim) Europeans tend to hold rather negative views of Muslims (Strabac and Listhaug, 2008; Savelkoul et al., 2012; Czymara, 2019). Part of anti-Muslim sentiments stems from general xenophobia (van der Noll and Saroglou, 2015). However, another driver of such views are concerns about religious fundamentalism (Helbling and Traunmüller, 2018). Tensions between Muslim and non-Muslim Europeans erupt in sad regularity, leading to violence against Muslims (Borell, 2015) as well as Islamist terror attacks in Europe (Jungkunz et al., 2018; Schmidt-Catran and Czymara, 2020). Knowing and understanding differences in values of Muslims and non-Muslims in Europe, hence, is crucial for peaceful coexistence. The fact that Europe's Muslim population is predicted to grow in the next years and decades (Pew Research Center, 2017) boosts the importance of establishing social cohesion within European societies. For a more insightful comparison, we tested differences in basic human values of Muslim immigrants in Europe compared to, first, non-religious native Europeans and, second, Christian native Europeans.

There are indeed significant differences in the distribution of human values among the three investigated groups. In line with prior research on the general role of religiosity for human values (Saroglou et al., 2004; Roccas and Elster, 2013), we find that Muslim immigrants are more likely to hold *conservation* related values (*tradition/conformity* and *security*) as well as the value *benevolence* compared to non-religious natives. Our results show that religiosity is positively correlated with *tradition*/*conformity* (which is true for Muslims and Christians). Moreover, differences in *tradition*/*conformity* between Muslims and the other groups disappear when accounting for differences in religiosity.

Contrary to theoretical expectations, however, Muslim immigrants in our sample agreed more to *universalism* than non-religious or Christian natives did. One reason for this unexpected finding could be experiences of discrimination and marginalization of Muslim immigrants in Europe. If this argument is true, then the higher agreement of Muslim immigrants to *universalism* would be the outcome of living under threat and in discrimination. In this case, integration would mean a decrease in the agreement to such values, approaching the levels of non-Muslim natives. While this has to remain speculation for the present study, future research based on longitudinal or time series data could shed more light on potential trends in values.

Although we see that the differences in values exist in all of the four analyzed countries, they are not all equal in size. We find that, in terms of human values, Muslims differ most strongly from Christians and non-religious natives in Sweden, while these differences are considerably weaker in Belgium, Germany, and France. The cross-national variation in the extent to which human values differ among the three groups can be explained by differences in experiences with religious diversity and ethnic co-existence, and by differences in national integration politics. Belgium and France have the longest national experiences with ethnic and religious diversity, dating back to, at least, the end of their colonial empires. In the case of France, the colonial empire to a large part covered areas with predominantly Muslim populations in Africa and the Middle East, many of which migrated to France after its collapse (Croucher, 2013; Kuru, 2016). In Sweden, in contrast, Muslim presence is just a couple of decades old (Bevelander and Otterbeck, 2010). It seems reasonable to hypothesize that cross-national differences in value gaps between Muslim immigrants and non-Muslim natives are at least in part due to the historical differences in dealing with immigration. However, value gaps in Germany were largely similar to those in Belgium in France. For a long time, Germany did not understand itself as an “immigration country” and it has less experience with integrating ethnic minorities into its host society (Ellermann, 2015). Hence, a national history of ethnic diversity seems to benefit integration outcomes, as France and Belgium demonstrate, but does not seem to be a necessary prerequisite, as Germany shows.

Integration politics that target stricter assimilation of ethnic and religious groups should encourage minorities to invest more into the social and cultural capital, ultimately leading values of immigrants to a converge to those of natives (Esser, 2010). Germany and France follow this approach more strongly (Koopmans, 2013). In contrast, Sweden has a very multi-cultural approach to integration, granting immigrants relatively easy access to equal rights and fewer incetintives for assimilation efforts regarding cultural or social capital (ibid). This could also explain why human values of Muslim immigrants and natives differ more in Sweden compared to Germany and France. However, Belgium also follows a multi-cultural approach to immigrant integration (ibid). Yet, value gaps in Belgium are more similar to those in France or Germany than to those in Sweden. Such politics, thus, do not seem to be an obstacle *per se* according to our data. In sum, neither a national history of diversity nor a country's integration politics alone are able to perfectly explain differences in human values of Muslim immigrants compared to natives. Our results thus suggest that it is a combination of both aspects that is most likely to be successful.

An alternative mechanism our study does not capture relates to national differences in inter-group relations. For example, Koopmans (2015) concludes that variation in Muslim fundamentalism among countries is the result of the level of fundamentalism of native Christians in the respective host society (p. 47). Our data does not allow a direct test of this hypothesis. However, the fact that Christian natives hold values that often tend to be more similar to non-religious natives compared to Muslim immigrants throughout all analyzed countries does not seem to support this reasoning. To test cross-national differences more thoroughly, we would need more countries in our data. Currently, few European countries exhibit a share of Muslims that is large enough for meaningful quantitative analyses.

One limitation of our study concerns sample size, the representativeness of the Muslim population and related generalizability of findings. While the ESS is a very high quality data source, it is not tailored to analyze ethnic or religious minorities in particular. This is shown by the low number of Muslims in the data, ranging from 2.29 percent of our analyzed sample in Sweden to 4.97 percent in Belgium (see Table 1). While the numbers are still sufficient to test for differences in our SEM, it limits the potential complexity of the model. This is reflected by the imperfect goodness of fit measures of our more complex models that take into account potentially relevant third variables. We pointed to the fact that the results of these models should, thus, be treated with some caution. Nevertheless, deeper analyses of the interplay between human values, religion and other characteristics could surely lead to interesting new insights. Perhaps even more problematic is that it is not perfectly clear how well the sampled Muslim population captures each country's actual Muslim population. It is particularly unlikely that less integrated and more fundamentalist Muslims participated in the survey, which might lead to an underestimation of group differences in our study. Programs that oversample immigrants or that offer questionnaires in origin country languages would be even more helpful in this respect. Unfortunately, we are not aware of such comparative data and especially none where the Schwartz values are included. The low number of Muslims in the sample also makes it impossible to differentiate between different generations (that is, being the son of immigrants or being an immigrant oneself). Surely, it would be interesting to see how values differ between the different generations of immigrants and if they become more similar to the host societies values with each generation (Drouhot and Nee, 2019).

Similarly, our analysis only includes four countries, which complicates a strict testing of any explanation for cross-national variation. The four countries we include differ in their immigration histories and their integration politics. Yet, many more countries would be needed to test these considerations more thoroughly. More observations on the country level would also allow examining alternative explanations, such as the importance of a country's pre-existing values. Sweden differs from the other countries also in its tradition of cultural liberalism and social democracy, making it particularly “WEIRD” (Schulz et al., 2019). This might be another reason why the contrast to Muslim immigrants, who come from less “WERID” countries, is most pronounced there. Unfortunately, the ESS does not include enough Muslim immigrants for most other countries for any quantitative analysis. Moreover, we would ideally need longitudinal data over decades to really make statements about over time developments, for instance regarding the importance of a country's past experiences. In these respects, we understand our study as a first step in understanding how social contexts shape values of ethnic and religious minorities in Europe—and why they may differ from those of natives.

Another issue is that being Muslim and being an immigrant (or, ultimately, an ethnic minority) is empirically strongly correlated in our data (see footnote 5). This does not come as a surprise given that none of the four countries under investigation has Islamic roots from a historical perspective. While some of the countries we examine have a longer Muslim tradition than others do, Muslim presence is still a relatively new phenomenon for all four countries, hardly exceeding two or three generations. This makes it hard to separate the impact of being Muslim from the impact of being a first or second generation immigrant. For an ideal comparison, we would need a group of Muslim natives in order to separate effects of being Muslim from effects of being immigrant. Unfortunately, such cases are really rare in our data. This may change in the future, when Muslim presence in Europe might be more established. Similarly, it would be highly interesting to examine the distribution of basic human values in predominantly Muslim societies outside Europe. Alexander and Welzel (2011) show that “glacial emancipative trends” can undermine public support for patriarchal values in Islamic societies. Similar trends might be observable regarding macro-level shifts in human values. To the best of our knowledge, unfortunately, large-scale data measuring human values outside of Western countries are currently not available.

Finally, our comparison of groups within different host countries does not allow disentangling origin effects from destination effects or community effects. That is, strictly speaking, we cannot say whether values of Muslim immigrants are imported from their countries of origin, shaped by certain conditions in the host country or result from the relations between origins and destinations. An ideal design would compare multiple origins in multiple destinations (see van Tubergen et al., 2004 for such a design testing the economic integration of immigrants). While existing comparative evidence on public attitudes and social norms shows significant differences between Muslims and non-Muslims in Western countries (Alexander and Welzel, 2011; Jäckle and Wenzelburger, 2015), our findings that value differences also vary between European countries suggests that country characteristics shape the integration processes, too. More thoroughly decomposing which aspects play a role could be an important step in understanding the cultural integration of ethnic and religious minorities in Europe.

## Data Availability Statement

The datasets generated for this study are available on request to the corresponding author.

## Ethics Statement

Ethical review and approval was not required for the study on human participants in accordance with the local legislation and institutional requirements. The patients/participants provided their written informed consent to participate in this study.

## Author Contributions

CC: conceptualization, data preparation, and writing original draft. ME: method and statistical analysis. CC and ME: editing final version. All authors contributed to the article and approved the submitted version.

## Conflict of Interest

The authors declare that the research was conducted in the absence of any commercial or financial relationships that could be construed as a potential conflict of interest.

## Footnotes

^1^We do not include the remaining values belonging to *openness to change* or *self-enhancement* in our analysis. This is because comparing all Schwartz values across three groups *and* all groups across four countries would make the study very complex, both in terms of theoretical expectations and in terms of empirical estimation. Not least, the computation of a model including all values is practically not feasible (see method section). Thus, we focus on those values, which we see as most relevant for our case. For a discussion on the relationship between the remaining values and religion in general, we refer to the review of Roccas and Elster (2013) and to the meta-analysis of Saroglou et al. (2004).

^2^Although religion and ethnicity are distinct concepts, both are interrelated in the case of European Muslims and religion is a central aspect of many ethnic identifications (Czymara, 2019, p. 6; also see Tibi, 2010; Brubaker, 2013).

^3^Note, however, that Koopmans (2015) reports that levels of religious fundamentalism among Muslims are high in Belgium.

^4^However, it is perhaps not too surprising that those who do not belong to a religion are significantly less religious compared to Muslims or Christians (see Table 1).

^5^It should be noted that being Muslim and being immigrant are almost perfectly correlated in our data, with only 3.6 percent of Muslims not having an immigration background (*n* = 67). Thus, we usually mean non-Muslims when we speak of “natives” in this article.

^6^We use MPlus Version 8 (Muthén and Muthén, 2017). All code is available at: https://dx.doi.org/10.17605/OSF.IO/YXR45.

^7^Fit measures [those below the threshold (see West et al., 2012) in italic]:

- Belgium: RMSEA = 0.048; PClose = 0.932; *CFI* = *0.932*; SRMR = 0.032
- France: RMSEA = 0.052; PClose = 0.104; *CFI* = *0.933*; SRMR = 0.035
- Germany: RMSEA = 0.050; PClose = 0.629; *CFI* = *0.938*; SRMR = 0.034
- Sweden: RMSEA = 0.056; *PClose* = *0.000*; *CFI* = *0.923*; SRMR = 0.038.

^8^We refrain from showing a similar table regarding the effects of age and education on each value due to reasons of space and since we do not have a strong theoretical interest in these relationships. The results are available upon request.

^9^Fit measures [those below the threshold (see West et al., 2012) in italic]:

- Belgium: RMSEA = 0.047; PClose = 0.996; *CFI* = *0.936*; SRMR = 0.031
- France: RMSEA = 0.048; PClose = 0.956; *CFI* = *0.940*; SRMR = 0.032
- Germany: RMSEA = 0.049; PClose = 0.709; *CFI* = *0.936*; SRMR = 0.033
- Sweden: RMSEA = 0.045; PClose = 1.000; *CFI* = *0.942*; SRMR = 0.035.
